# The Study Influence Analysis of the Mathematical Model Choice for Describing Polymer Behavior

**DOI:** 10.3390/polym15173630

**Published:** 2023-09-01

**Authors:** Anna A. Kamenskikh, Yuriy O. Nosov, Anastasia P. Bogdanova

**Affiliations:** Department of Computational Mathematics, Mechanics and Biomechanics, Perm National Research Polytechnic University, 614990 Perm, Russia; ura.4132@yandex.ru (Y.O.N.); anstasia_pankova@mail.ru (A.P.B.)

**Keywords:** Hertz contact, elastic model, elastic–plastic model, viscoelastic model, polymer, deformation behavior, contact, contact pressure, Prony model, gamma-modified PTFE

## Abstract

The article considered the three types of description of the material behavior model: elastic, elastic–plastic, and viscoelastic. The problem is considered in the framework of deformable solid mechanics. The paper considers the possibility of describing modern polymeric and composite materials used as antifriction sliding layers in the viscoelasticity framework. A numerical procedure for finding the coefficients to describe the viscoelastic material behavior using the Prony model has been implemented. Numerical results and experimental data are compared. The model problem of spherical indenter penetration into polymer half-space is realized. The influence of the system discretization on the numerical solution is analyzed. The influence of the polymer behavior description in static and dynamic problem formulations is analyzed.

## 1. Introduction

### 1.1. Research Objectives

The investigate different types of mathematical models of behavior describing of a polymer material is the purpose of the work: elasticity; elastoplasticity; viscoelasticity. As well as to evaluate the possibility of applying the model depending on the type of analysis and load. 

Research objectives:

1. The behavior description of polymeric materials in viscoelastic terms using the generalized Maxwell model;
2. Comparison of experimental data and numerical solutions of three different mathematical models of material behavior: elastic, plastic, and viscoelastic;
3. Carrying out verification of the numerical Hertz problem;
4. Constructing numerical models of the specimen behavior (elastic–plastic, viscoelastic);
5. The influence study of mathematical models of polymers’ behavior in a dynamic setting (loading and subsequent exposure for 1 h at room temperature).

### 1.2. Problem Context

The study of various characteristics of materials, as well as their application possibility in this or that field, is an urgent task among scientists all over the world [1,2,3]. One of the modern popular materials is polymers. Such materials are useful because, with the help of certain combinations of their structural links, it is possible to obtain materials with different characteristics [4]. In all production areas, there is a search for a type of polymeric material that will have all possible positive characteristics but, at the same time, will have low cost and a low environmental footprint, both in manufacturing and disposal [5,6]. Scientists from all over the world create certain combinations of monomers and further investigate their characteristics and properties [7]. One important quality of polymers is the combination of various useful properties, such as impact resistance, electrical conductivity, and insulation [8], plasticity and elasticity, lightness, chemical resistance, impermeability, corrosion resistance, etc.

In particular, one of the actively used polymeric materials in industry is PTFE, along with its various modifications [9,10]. It is a material that has unique properties such as high chemical resistance, low thermal conductivity, abrasion, and corrosion resistance, and high dielectric constant. Both pure PTFE and composites based on it are used in various industries, such as mechanical engineering, the oil and gas industry, electrical engineering, medicine, etc. PTFE and its modifications are widely used in the production of electronic components and equipment such as capacitors, resistors, switches, and other components since they have a high dielectric constant and high temperature resistance [11,12,13].

In the chemical industry, manufacturers and researchers use PTFE as a gasket and rod material [14]. PTFE is a biocompatible material and has no allergic reactions. Therefore, it has wide application in medicine for the production of prosthetic joint implants, prostheses, catheters, and other medical devices [15,16]. In mechanical engineering, PTFE and composites based on it are used in the production of bearings, shafts, seals, and other components that must operate under conditions of high temperature and pressure [17,18,19]. In its pure form, PTFE has a number of characteristics; however, its essential feature is its bonding with reinforcing elements. Fluoroplastic is mainly used as a matrix to create a new material. In particular, its gamma radiation modifications are the matrix for a wide range of composite materials [20]. In numerical simulation of the performance of polymeric materials and composites based on them, an important step is the correct choice of the approach to describe both the material’s properties and its behavior [21]. There are different approaches to building qualitative models of composites. One of them is to describe the properties of the matrix and the inclusions or reinforcing elements separately from one another [22,23].

Studies of the properties of polymers and composites based on them have existed since the appearance of polymers themselves. However, much of this research has focused on the study of these materials as elastic or elastic–plastic bodies. It was found that the data obtained in solving such problems are not sufficient to qualitatively describe the behavior of the material. This is due not only to the lack of description of viscous behavior but also to the presence of a description of plasticity. It is noted that polymers are generally divided into two types: viscoelastic–plastic and viscoelastic. In addition, if, in the first type there, is a trace of plastic deformations, the second type will have insignificant plastic deformations. Studies aimed at analyzing both the viscoelastic–plastic behavior of materials [24,25,26,27] and viscoelastic behavior of materials [28,29,30] are gaining momentum. The paper considers a mathematical model of only viscoelastic behavior for the first stage of the study.

### 1.3. Problem Description

Polymer materials have gained a strong foothold in the international market and are actively used on a large scale in various industrial fields. In particular, the materials are used as sliding layers in various types of friction units. However, there is a lack of research on the deformation behavior of polymers under various design conditions [31,32,33]. In particular, studies are most often directed towards a single configuration of a working structure. However, in order to qualitatively describe the behavior of the structure, it is necessary to consider its operation in a complex [34]. For this purpose, it is necessary to consider the problem not in a static formulation, but in a dynamic one, taking into account such factors as variable cyclic load, variable temperature, and different variations of geometrical configuration. However, to qualitatively describe and consider all these factors, it is necessary to choose the right description of the material behavior model.

This work aims to investigate the influence of the choice of a polymer behavior model. Gamma-modified PTFE is described in three ways: an elastic body, an elastic–plastic body, and a viscoelastic body. The model problem of introducing a spherical indenter into polymer half-space is realized.

## 2. Materials and Methods

### 2.1. Materials

Composite materials are widely spread in different spheres of human activity. However, creating and study the matrix [35,36] and composite fillers [37,38] is an important task. Matrices from different materials allow the structure to work in different conditions: increased and decreased temperatures [39], responsible friction nodes [40], aggressive environments [41], etc.

**Figure 1:**
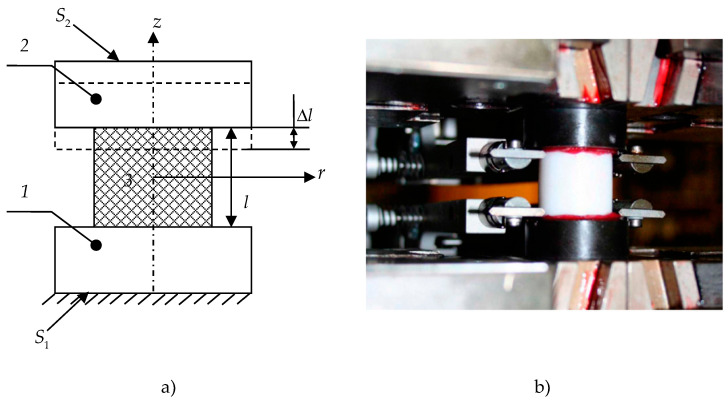
Experimental studies: (**a**) experiment scheme; (**b**) full-scale sample. Sections 1 and 2 are upper and lower steel sections, respectively; 3—polymer.

The article presents a study of one of the common matrices of composite materials in bridge-building activities—gamma-modified PTFE. The material has high strength and antifriction properties [42]. A full-scale experiments series were realized to determine the following: elastic characteristics, stress–strain dependence, friction properties, and dynamic characteristics for this material. Experimental studies were performed by Dr. Adamov A.A. using the equipment of the Ural Branch of the Russian Academy of Sciences. The experimental study was conducted using Zwick Z100SN5A (Zwick Roell AG, Ulm, Germany), which allows mechanical, quasi-static tests for uniaxial tension, compression, creep, etc. Test specimens are made in the form of cylindrical bodies (Figure 1) with characteristic dimensions: length, l=20±0.1 mm; radius, r=10±0.1 mm.

The specimen is deformed by Δl up to 2 mm from the original size. The surface $S_{1}$ is rigidly fixed. Displacements are applied at a constant rate $V_{\Delta l}$ = 0.006 mm/min on the surface $S_{2}$. The paper has established that the material has no barrel-like deformation during experimental investigations. Consequently, only is realized in the z-axis direction within the experiment.

The elastic compression modulus E=863.8 MPa was determined within the experiment at uniaxial deformation under constrained compression. The Poisson’s coefficient ν=0.461 was determined using uniaxial deformation experiment.

**Figure 2:**
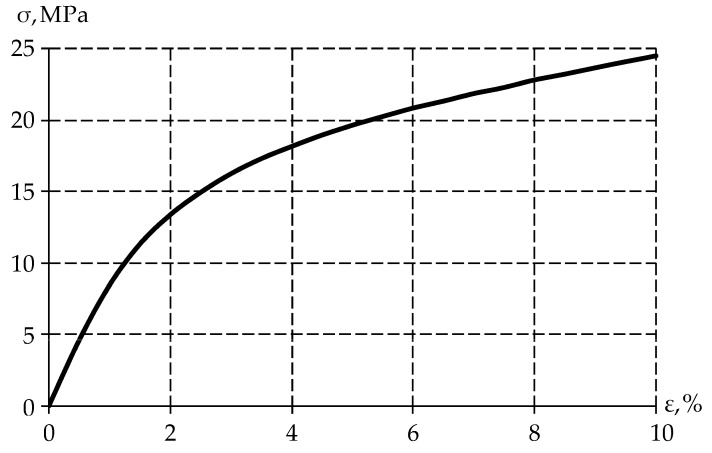
The σ−ε diagram for gamma-modified PTFE.

The specimen was uniaxial compression stress tested (ε=10%) to obtain the stress–strain relationship at (Figure 2).

The figure shows that the PTFE elastic behavior occurs when the sample is deformed up to 1%, after which the material enters plasticity. It was also observed that the material recovered its original geometric dimensions after some time. Therefore, plastic deformation is negligible in the polymer. From this, it can be concluded that the stress reduction is due to the viscoelastic behavior of the material.

The next step was to determine the dynamic characteristics of the material under uniaxial stress. One loading cycle was carried out:

- The specimen was tested at a strain rate of 0.006 mm/min up to 10%;
- Constant strain was held for 15 min;
- The load was removed from the material until normal stresses of 0.1 MPa was reached at a rate of 0.006 mm/min.

**Figure 3:**
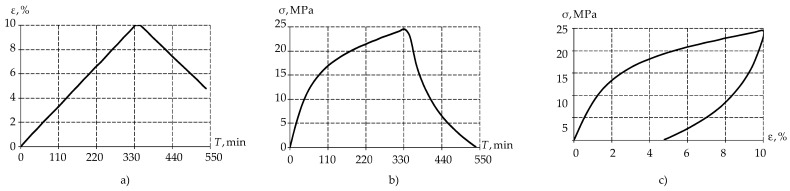
Dynamic characteristics determination of the material: (**a**) strain–time dependence, (**b**) stress–time dependence; (**c**) stress–strain dependence.

Based on this, the following dependencies were obtained: strains on time (Figure 3a), normal stresses on time (Figure 3b), and stresses on strain (Figure 3c).

The obtained data will be used to describe the material behavior via the deformation theory of elastic–plasticity and the model of viscoelastic behavior based on the Prony series.

### 2.2. Description of Polymer Behavior

Currently, three types of material description are common: an elastic body, an elastic–plastic body [43], and a viscoelastic body [44]. The elastic–plastic body is described by the deformation theory of elastic–plasticity [45].

Many authors consider polymeric materials, matrices, and composites in the form of a Maxwell body [46,47]. The Prony series is the most common model for describing a Maxwell body [48].

The mathematical formulation of the problem includes the equilibrium equation:

(1) $$\mathrm{div}\;\hat{\sigma }=0,$$

where $\hat{\sigma }$ is the stress tensor.

The problem is considered in the framework of large deformations:

(2) $$\hat{\varepsilon }=\left[\nabla \bar{u}+{\left(\nabla \bar{u}\right)}^{T}+\nabla \bar{u}{\left(\nabla \bar{u}\right)}^{T}\right]/2,$$

where $\bar{u}$ is the displacement vector; and $\hat{\varepsilon }$ is the strain tensor.

Let us write down the stress–strain relationship for each case when describing the polymer material by different models:

- Elastic body:

(3) $$\hat{\sigma }=\lambda I_{1}\left(\hat{\varepsilon }\right)\hat{I}+2\mu \hat{\varepsilon },$$

where λ and μ are Lame parameters; $I_{1}\left(\hat{\varepsilon }\right)$ is the first invariant of the stress tensor; and $\hat{I}$ is a unit vector;

- Elastoplastic body:

(4) $$\hat{\sigma }=2\sigma_{I}\left[\hat{\varepsilon }-I_{1}\left(\hat{\varepsilon }\right)\hat{I}/3\right]/\left(3\varepsilon_{I}\right)+KI_{1}\left(\hat{\varepsilon }\right)\hat{I},$$

where $\sigma_{I}$ and $\varepsilon_{I}$ are the stress and strain intensity, respectively; and K is the bulk modulus of elasticity;

- Viscoelastic body:

(5) $$\hat{\sigma }=\int_{0}^{T}\left[E_{\infty }+E_{0}\sum_{i=1}^{k}\alpha_{i}\exp\left(-\left(T-\tau \right)/\beta_{i}\right)\right]d\varepsilon \left(\tau \right),$$

where $E_{0}$ and $E_{\infty }$ are Young’s moduli at the initial and final moment of time, respectively; $\alpha_{i}$ is weighting coefficient; and $\beta_{i}$ is relaxation times.

**Figure 4:**
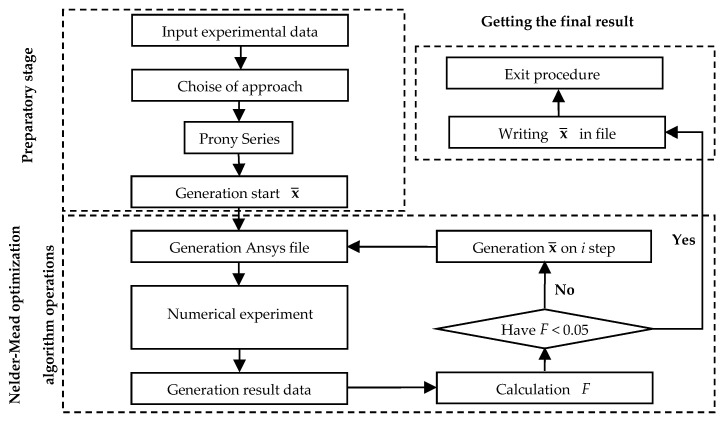
The numerical procedure scheme.

A numerical procedure is created to describe the viscoelastic behavior of a polymer material (the scheme is presented in Figure 4). Experimental data are input to this procedure. The unknown coefficients are found by solving the minimization problem of function (6). The presented functional has a parabolic form. Therefore, it has one global minimum (one solution). They are necessary for a correct description of material behavior.

(6) $$F=\sum_{j=1}^{n}{\left[\sigma_{j}^{Exp}-\sigma_{j}^{Num}\left(\bar{x}\right)\right]}^{\;2},$$

where n is the number of experimental points; $\sigma_{j}^{Exp}$ is the experimental stress value at j point; $\sigma_{j}^{num}\left(\bar{x}\right)$ is the numerical stress value at j point; and $\bar{x}=\left(\alpha_{i},\beta_{i}\right)$ is the vector of unknowns, which consists of 2 k parameters.

This numerical procedure is used to find the coefficients necessary to describe the viscoelastic behavior of the material. The solution is realized using the finite element method in ANSYS Mechanical APDL 2021R2 (Livermore, California, USA) software package in synergy with Python. During the operation of the numerical procedure, the coefficients are selected, then the experiment is numerically realized, and the results of numerical and in situ experiments are verified. The numerical procedure runs until the error is less than 5% between the experimental and numerical values.

The numerical procedure consists of 3 stages:

- First (preliminary) stage: input of experimental data in the form of a text file with data, model selection, and generation of the initial vector of unknowns from Equation (5);
- The second stage is based on the Nelder–Mead optimization algorithm: creation of a script-file describing the numerical experiment in ANSYS Mechanical APDL, conducting the numerical experiment, obtaining the results file, comparison of numerical and experimental data, generation of the vector of unknowns from Equation (5) at step i, and transition to the next iteration;
- The third (and final) step is performed when the error between the experimental and numerical data reaches 5%: formation of the final vector of unknowns from Equation (5) and exit from the procedure.

### 2.3. The Hertz Formulation

**Figure 5:**
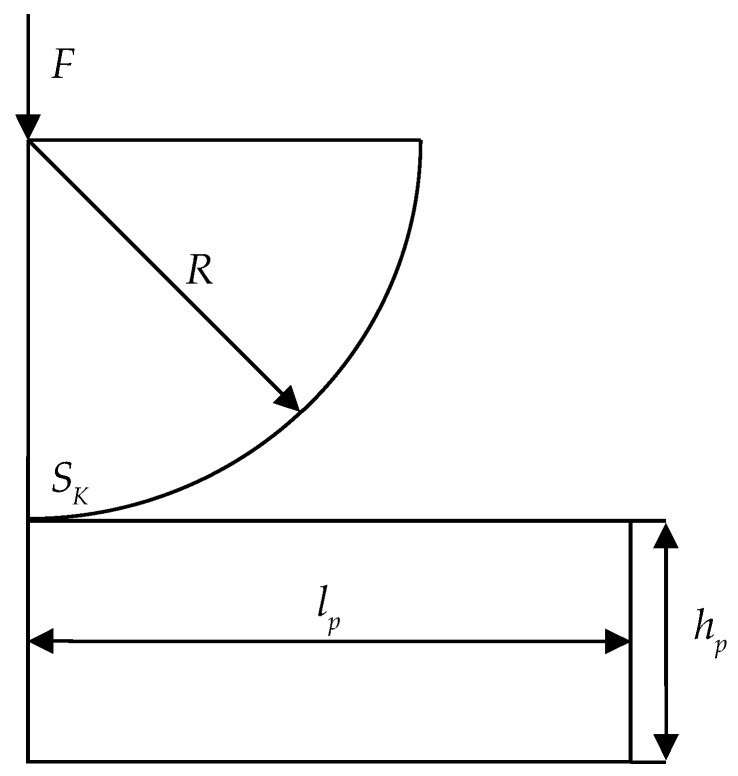
Scheme of the Hertz contact problem.

The Hertz contact problem is one of the common problems of contact interaction between an indenter and a half-space [49]. Figure 5 shows the contact interaction scheme between a spherical indenter and a half-space.

The spherical indenter of radius R=0.2 m is penetrated with force F=1000 N into a half-space with geometrical characteristics: length is $l_{p}=0.31$ m; height is $h_{p}=0.05$ m.

All possible contact states at the site $S_{K}$ are considered within the problem. The contact boundary conditions are of the following forms:

- Sliding friction: $u_{n}^{1}=u_{n}^{2}$, $u_{\tau_{1}}^{1}\neq u_{\tau_{1}}^{2}$, $u_{\tau_{2}}^{1}\neq u_{\tau_{2}}^{2}$, $\sigma_{n}^{1}=\sigma_{n}^{2}$, $\sigma_{n\tau_{1}}^{1}=\sigma_{n\tau_{1}}^{2}$, $\sigma_{n\tau_{2}}^{1}=\sigma_{n\tau_{2}}^{2}$, when $\left|\sigma_{n\tau_{1}}\right|=\mu \left(\sigma_{n}\right)\left|\sigma_{n}\right|$;
- No contact: $\left|u_{n}^{1}-u_{n}^{2}\right|\geq 0$, $\sigma_{n\tau_{1}}=\sigma_{n\tau_{2}}=\sigma_{n}=0$;
- Adhesion: ${\bar{u}}^{1}={\bar{u}}^{2}$, $\sigma_{n}^{1}=\sigma_{n}^{2}$, $\sigma_{n\tau_{1}}^{1}=\sigma_{n\tau_{1}}^{2}$, $\sigma_{n\tau_{2}}^{1}=\sigma_{n\tau_{2}}^{2}$,

where $\mu \left(\sigma_{n}\right)$ is friction coefficient; $\tau_{1}$ and $\tau_{2}$ are the axes designations that lie in the plane tangent to the contact surface; $u_{n}$ are displacements along the normal to the corresponding contact boundary; $u_{\tau_{1}}$ and $u_{\tau_{2}}$ are displacements in the tangent plane; $\sigma_{n}$ is stress along the normal to the contact boundary; $\sigma_{n\tau_{1}}$ and $\sigma_{n\tau_{2}}$ are tangential stresses at the contact boundary; $\sigma_{n\tau }$ is the value of the tangential contact stress vector; and 1 and 2 are conditional numbers of the contacting surfaces.

The Hertz solution is obtained for the case of a parabolic pressure profile and has the following form:

(7) $$p\left(r\right)=p_{0}{\left(1-r^{2}/a^{2}\right)}^{1/2},$$

where r is the distance for an arbitrary point on the plane; a is the Hertz contact radius; and $p_{0}$ is the maximum contact pressure.

This dependence will be used to analyze the convergence of the problem within the static problem formulation in a subsequent study.

## 3. Results

### 3.1. Invastigation of Mathematical Models

The mathematical model choice for describing material behavior is an important part of computer engineering as it affects the accuracy of the results obtained in the study. This article compared experimental data and mathematical models at the first stage of the study.

**Figure 6:**
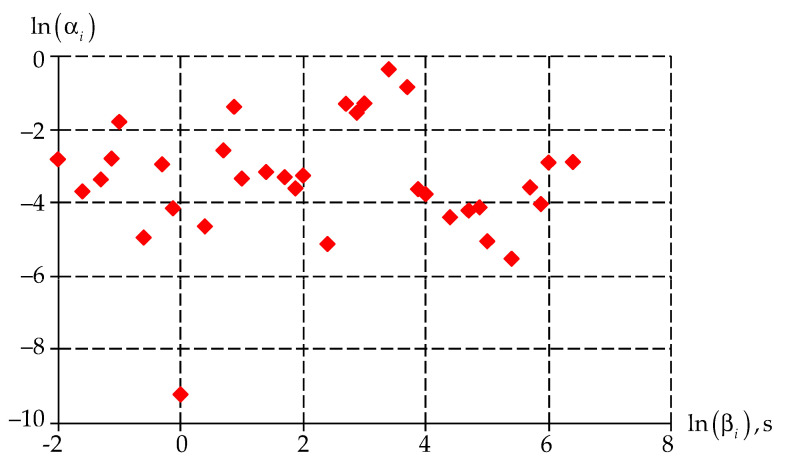
Dependence of weight coefficients $\alpha_{i}$ on relaxation time $\beta_{i}$. Red dots are the value of the weight coefficient $\alpha_{i}$ at a certain relaxation time $\beta_{i}$.

The numerical procedure (Section 2.2) allows us to find the vector of unknowns for the Prony series with an error of less than 5%. The final vector of unknowns is presented in Figure 6.

It can be noted that the weight coefficients $\alpha_{i}$ have the largest values in the values range of relaxation times $\beta_{i}$ [$10^{2}$;$10^{4}$].

**Figure 7:**
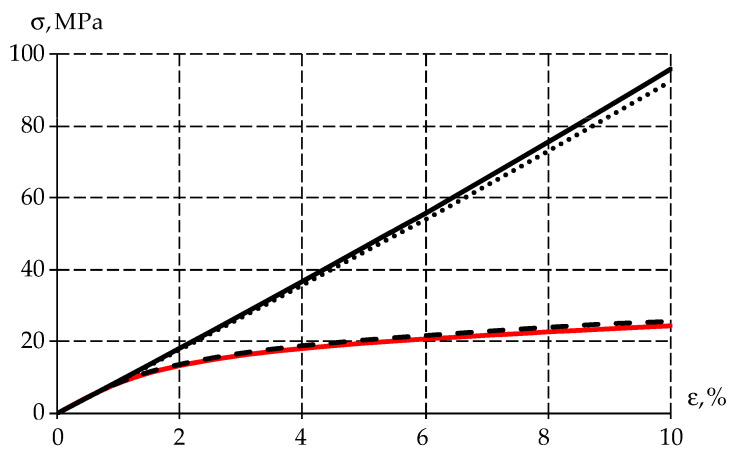
Stress–strain diagram: the red line is experimental data; the black solid line is the elastic body; the dashed line is the elastic–plastic body; and the dots are the viscoelastic body.

Numerical modeling of a cylindrical specimen uniaxial deformation has been conducted—similarly to the field experiment (paragraph 2.1.). Uniaxial deformation occurs up to a strain value equal to 10%. The problem was solved in a static formulation and let us obtain the stress–strain diagram for all considered variants of body behavior (Figure 7).

Elastic and viscoelastic bodies behave linearly over the entire deformation interval of the specimen when solving the static problem. At the same time, the elastic–plastic body describes the deformation of the specimen up to 10%, with an error of less than 5%. Consequently, the use of the mathematical model of the elastic–plastic body allows us to explore problems in analyzing the strength of a structure within the framework of static calculations.

**Figure 8:**
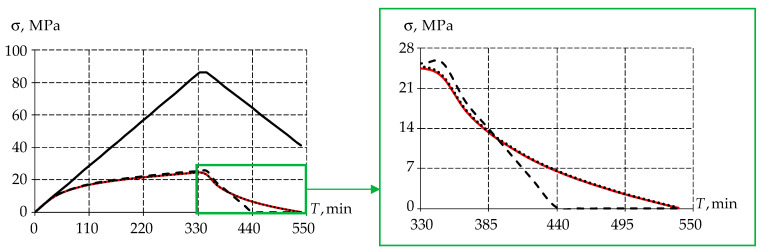
Stress dependence on time: the red line is experimental data; the black solid line is the elastic body; the dashed line is the elastic–plastic body; the dots are the viscoelastic body.

Moreover, predicting the performance of the structure at all stages of its life cycle is an important feature of numerical modeling. Let us perform numerical modeling of the experiment depending on the time of the load impact on the specimen (Figure 8).

The article notes that when comparing the numerical solution with the experimental one, it does not fully describe the behavior of the material and has a linear character over the entire range of deformation in the framework of the theory of elasticity. In the framework of elastic–plasticity and viscoelasticity theory, the time dependencies of stresses present a more qualitative description of material behavior. The difference from the experimental data in the elastic–plastic model is, at the loading stage, 5%, and at the unloading stage, 20%. This is due to significant plastic deformations, due to which there is an extreme drop in stress. In the case of the viscoelastic body the difference from experimental data is, at loading stage, 1%, and at the unloading stage, 0.53%. Consequently, the description of polymer behavior by the viscoelastic model allows for a better description of its dynamic behavior.

**Figure 9:**
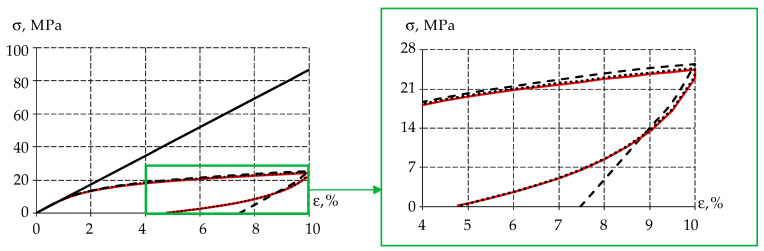
Stress dependence on deformation: the red line is experimental data; the black solid line is the elastic body; the dashed line is the elastic–plastic body; and the dots are the viscoelastic body.

For a qualitative assessment of the material behavior, we show the stress–strain dependence in Figure 9.

Similar to the time dependence of stresses, a significant difference between the elastic body and all others is noted. The elastic body behaves linearly during the loading and unloading phases of the numerical model. It should be noted that for an elastic–plastic body at unloading, the stress values decrease linearly, which is due to the accumulated plastic deformation in the body. There is no zone of stress reduction at load holding. When considering the viscoelastic model, it is worth noting the qualitative and quantitative description of material behavior in the dynamic formulation.

The following conclusions can be drawn from the above: it is necessary to use the elastic–plastic model to analyze the structure strength in the static setting, and it is necessary to use the viscoelastic model to predict the performance of the structure during the life cycle.

### 3.2. Hertz Contact Calculation Model

The model problem of indenter penetration into a half-space is solved to practice the use of mathematical models in the structure operation.

**Figure 10:**
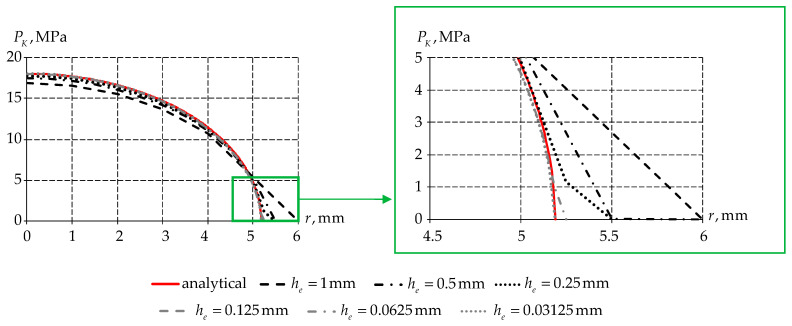
Analysis of finite element partitioning of the Hertz contact.

The first study step is to determine the optimal value of the mesh size within the framework of computer engineering. For this purpose, we conduct a series of numerical experiments to determine the optimal finite element partition size (Figure 10). The size of the finite element near the contact is chosen as the variable parameter $h_{e}$.

When analyzing the finite element partitioning, it can be seen that the largest error occurs in the leftmost and rightmost contact nodes. However, when the finite element size is reduced, the numerical solution approaches (7). An error of less than 1% is achieved with finite element partitioning $h_{e}=0.03125\;\mathrm{mm}$.

**Figure 11:**
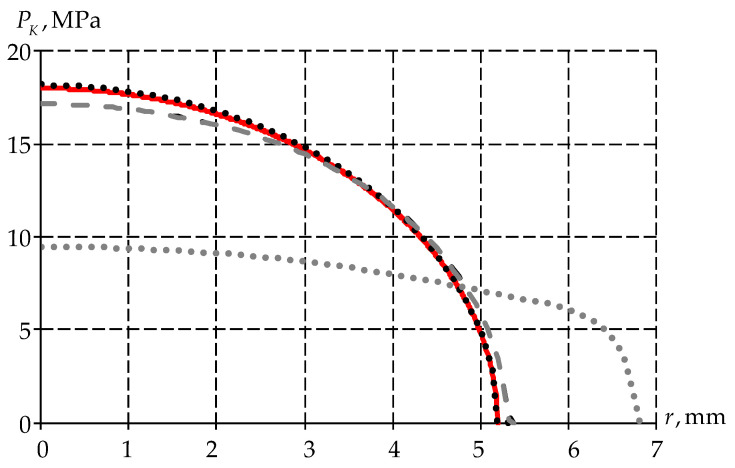
Dependence of contact pressure on the radius of indenter insertion: the red line is the analytical solution; black lines are maximum load; gray lines are 1 h exposure time; the dashed line is the elastic–plastic body; the dots are the viscoelastic body.

Further, we realize the problem in dynamic formulation for two models: the elastic–plastic body and the viscoelastic body. The paper also considers the value of contact pressure at maximum load and its endurance for 1 h (Figure 11).

An elastic–plastic body does not change with time. In a viscoelastic body, the following are observed: a decrease in contact pressure values, and an increase in the contact area between the spherical indenter and the half-space.

**Figure 12:**
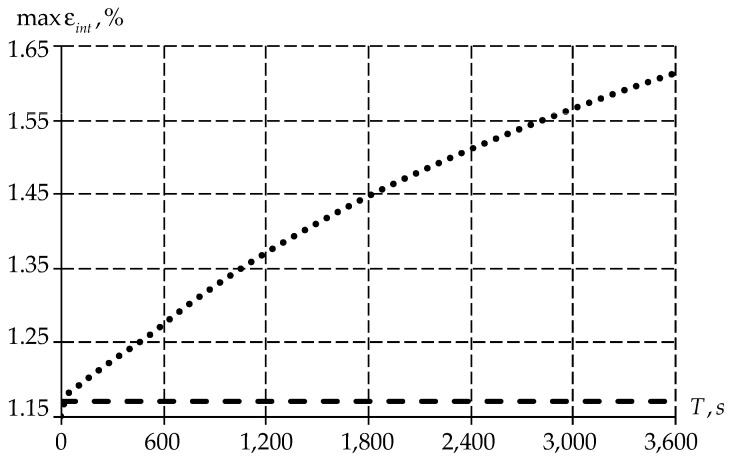
Dependence of maximum values of strain intensity on time: the dotted line is the elastic–plastic body; the dots are the viscoelastic body.

Further, the article considers the distribution of maximum strain values as a function of time (Figure 12).

It should be noted that the initial point of the strain intensity plots coincide for elastic–plastic and viscoelastic bodies. However, with the time passage, the values of strain intensity in the elastic–plastic body are constant. At the same time, the value of the viscoelastic body grows nonlinearly. From the above, we can conclude that the creep of the material occurs with the passage of time.

**Figure 13:**
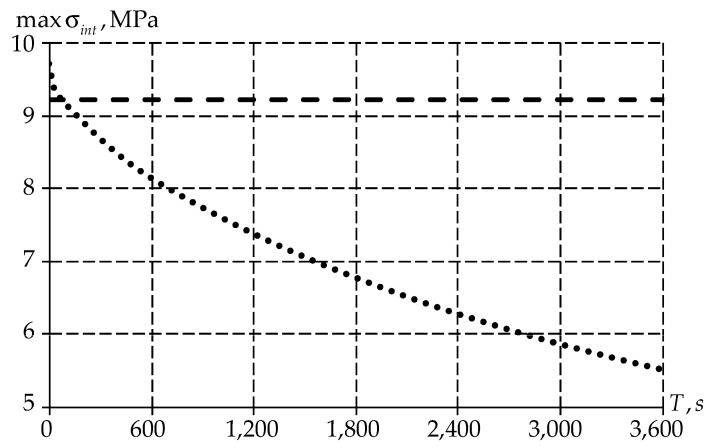
Dependence of maximum values of stress intensity on time: the dotted line is the elastic–plastic body, the dots are the viscoelastic body.

Let us also consider the maximum values distribution of stress intensity (Figure 13).

Similar to deformations, the stress intensity has a different distribution of values over time depending on the body type. An elastic–plastic body has a constant value over the entire time range. At the same time, for a viscoelastic body, the level of stress intensity decreases with time due to the material creep (Figure 12).

## 4. Discussion

### 4.1. Limitation Statement

The paper presents the results of numerical identification and simulation of the behavior of gamma-modified PTFE. The work has a number of limitations that are planned to be eliminated in the future:

1. The material behavior is considered at a constant temperature of 20 °C;
2. The model problem of spherical indenter penetration into a half-space is considered;
3. For each material, it is necessary to carry out a separate description of the mathematical model;
4. Long time ranges are not considered, while the material works for a long time.

Further directions for the development of the work:

1. Investigation of the material on a large range of operating temperatures;
2. Study of the material on the dependence on the load impact rate on the polymer material;
3. Study of temperature characteristics of the material;
4. Realization of the problem on the example of a bridge bearing structure under cyclic loading.

In the future, we plan to proceed to consideration of a real structure within the framework of contact interaction problems. The real structure consists of two steel plates: one with a spherical indenter and one with a spherical notch, and there is a sliding layer between them. The Hertz model was chosen as a simplified model for the initial verification of the obtained results on the viscoelastic behavior of the polymer material.

When considering the model of a real structure, we plan to simulate its operation under cyclic loads, as well as at different temperature parameters. In this case, we plan to obtain a description of deformation behavior as close as possible to the behavior of a real structure in which irreversible deformations occur over time.

### 4.2. On the Choice of a Mathematical Model

There is a certain variety of descriptions for the viscoelastic–plastic behavior of a material; in particular, there are such models as the cooperative-viscoplasticity theory based on overstress (VBO) model [16], combining a nonlinear viscoelastic model with a viscoplastic model using the von Mises yield criterion [12,24,50,51,52], etc. However, in the present work, gamma-modified PTFE is considered. In a number of experiments, it is noted that there is no need to describe the behavior of the material as a viscoelastic–plastic body; a viscoelastic model is sufficient for a qualitative description of its behavior. Within the framework of the first approximation, the Prony viscoelastic model was chosen.

As an example, consider the work [53], in which an experimental and numerical study of a Prony series as the main relation of the matrix description was conducted. It is shown that when the number of unknown terms increases, the numerical data describe the experimental data with an error of 10%.

This model has a number of advantages: a sufficient description of material behavior, the active application of this model by other researchers [29,54], the relative simplicity of the mathematical description, etc. The numerical algorithm has been tested early for the description of lubricant behavioral models [55].

### 4.3. Applicability of the Research

Gamma-modified PTFE is widely used in bridge bearings as an antifriction material [40,45,56]. In this design, the material operates under large temperature differences (from −40 °C to +40 °C) and cyclic loads directed in different directions; thus, numerical experiments in dynamic formulation are necessary for qualitative prediction of the load-bearing structure performance. It was obtained that the elastic–plastic description of materials is suitable only for describing the performance of the structure in the static formulation; thus, to describe the material behavior over time, it is necessary to describe it in a viscoelastic formulation. In the future, it is planned to transfer to a model of a real bridge support structure with a different set of temperature and cyclic tests.

## 5. Conclusions

Description of material behavior is an important part of computational engineering research. Its correct description allows us to qualitatively predict the performance of a structure during its life cycle. Within the framework of this work, gamma-modified PTFE has been described in three ways: an elastic body, an elastic–plastic body, and a viscoelastic body. Within the numerical analysis, the following has been established:

- The use of an elastic–plastic body to describe the material behavior can be used only in static problems to determine the strength properties of the structure;
- The use of a viscoelastic body to describe the mathematical model of material behavior allows for evaluation of the performance of a structure at the entire stage of its life cycle.

The model problem of introducing a spherically shaped indenter into polymer half-space is realized.

The research presented in the article will draw attention to the necessity of correct and detailed descriptions of mathematical models of material behavior. This, in turn, will allow for improvements to the quality of solved problems within the computer engineering framework. At the same time, at early stages, it will be possible to track problem areas of the structure operation and perform manipulations for their elimination in a timely manner.

The presented study can be used in compression contact nodes; for realization in tensile problems, it is necessary to undertake additional research.

## Data Availability

Not applicable.
